# Altered expression of microRNA-223 in the plasma of patients with first-episode schizophrenia and its possible relation to neuronal migration-related genes

**DOI:** 10.1038/s41398-019-0609-0

**Published:** 2019-11-11

**Authors:** Zhilei Zhao, Seiichiro Jinde, Shinsuke Koike, Mariko Tada, Yoshihiro Satomura, Akane Yoshikawa, Yukika Nishimura, Ryu Takizawa, Akihide Kinoshita, Eisuke Sakakibara, Hanako Sakurada, Mika Yamagishi, Fumichika Nishimura, Aya Inai, Masaki Nishioka, Yosuke Eriguchi, Tsuyoshi Araki, Atsuhiko Takaya, Chiemi Kan, Maki Umeda, Akihito Shimazu, Hideki Hashimoto, Miki Bundo, Kazuya Iwamoto, Chihiro Kakiuchi, Kiyoto Kasai

**Affiliations:** 1 Department of Neuropsychiatry, Graduate School of Medicine, the University of Tokyo, Bunkyo-ku, Tokyo, 113-8655 Japan; 2 International Research Center for Neurointelligence, The University of Tokyo, Bunkyo-ku, Tokyo, 113-0033 Japan; 3 Department of Child Neuropsychiatry, Graduate School of Medicine, the University of Tokyo, Bunkyo-ku, Tokyo, 113-8655 Japan; 4 Department of Psychiatry, Fukui Kinen Hospital, Miura City, Kanagawa 238-0115 Japan; 5 Department of Mental Health, Graduate School of Medicine, the University of Tokyo, Bunkyo-ku, Tokyo, 113-0033 Japan; 6 Department of Public Health Nursing, Graduate School of Nursing Science, St. Luke’s International University, Chuo-ku, Tokyo, 104-0044 Japan; 7 Center for Human and Social Sciences, College of Liberal Arts and Sciences, Kitasato University, Sagamihara City, Kanagawa 252-0373 Japan; 8 Department of Health Economics and Epidemiology Research, School of Public Health, the University of Tokyo, Bunkyo-ku, Tokyo, 113-0033 Japan; 9 Department of Molecular Brain Science, Graduate School of Life Sciences, Kumamoto University, 1-1-1 Honjo, Kumamoto City, Kumamoto, 860-8556 Japan

**Keywords:** Schizophrenia, Molecular neuroscience

## Abstract

Recent studies have shown that microRNAs (miRNAs) play a role as regulators of neurodevelopment by modulating gene expression. Altered miRNA expression has been reported in various psychiatric disorders, including schizophrenia. However, the changes in the miRNA expression profile that occur during the initial stage of schizophrenia have not been fully investigated. To explore the global alterations in miRNA expression profiles that may be associated with the onset of schizophrenia, we first profiled miRNA expression in plasma from 17 patients with first-episode schizophrenia and 17 healthy controls using microarray analysis. Among the miRNAs that showed robust changes, the elevated expression of has-miR-223-3p (miR-223) was validated via quantitative reverse transcription-polymerase chain reaction (qRT-PCR) using another independent sample set of 21 schizophrenia patients and 21 controls. To identify the putative targets of miR-223, we conducted a genome-wide gene expression analysis in neuronally differentiated SK-N-SH cells with stable miR-223 overexpression and an in silico analysis. We found that the mRNA expression levels of four genes related to the cytoskeleton or cell migration were significantly downregulated in miR-223-overexpressing cells, possibly due to interactions with miR-223. The in silico analysis suggested the presence of miR-223 target sites in these four genes. Lastly, a luciferase assay confirmed that miR-223 directly interacted with the 3′ untranslated regions (UTRs) of all four genes. Our results reveal an increase in miR-223 in plasma during both the first episode and the later stage of schizophrenia, which may affect the expression of cell migration-related genes targeted by miR-223.

## Introduction

Schizophrenia is one of the most common mental disorders, affecting ~1% of the worldwide population. The onset of this disease often occurs during adolescence, and the subsequent chronic course of the illness can become a heavy burden and a serious public health issue^1^. Therefore, there is an urgent need to elucidate the pathophysiology of the early stages of schizophrenia. Although the exact cause of this disease remains obscure, decades of intensive studies have indicated that various mechanisms of gene regulation, including those employed by noncoding RNAs, participate in the etiology of schizophrenia.

MicroRNAs (miRNAs) are short noncoding RNAs (19–23 nucleotides) that regulate gene expression at the transcriptional and posttranscriptional levels through interaction with the 3′ untranslated region (UTR) of target messenger RNA (mRNA)^2–4^. Emerging evidence has suggested that over 50% of known miRNAs are expressed in the central nervous system, where they play a crucial role in the regulation of neurodevelopment, synaptogenesis, and synaptic function^5–9^; this evidence suggests their active involvement in the development of psychiatric diseases. Specifically, recent studies on postmortem brains from patients with schizophrenia have shown alterations in the expression levels of specific miRNAs^10–18^. Other studies have reported that circulating miRNAs, which were detected in several body fluids, including serum, plasma, and cerebrospinal fluid, are also influenced by the pathological conditions of many psychiatric disorders, including schizophrenia^19–22^. Despite the accumulating evidence described above, the changes in plasma miRNA levels that occur at the initial stage of schizophrenia have not been fully explored.

In the present study, using a global screening approach, the altered expression profiles of miRNAs in plasma from patients with first-episode schizophrenia (FES) were investigated. To further provide a possible molecular explanation for the altered miRNA expression, we also explored the putative target mRNAs of the candidate miRNA.

## Materials and methods

### Participants

A total of 76 Japanese individuals (all of Asian ethnicity) participated in the present study. In the first set, 17 outpatients who were diagnosed with FES (as described below) and 17 age- and sex-matched control subjects were included (Table 1). Furthermore, an independent second set consisting of 21 inpatients with schizophrenia and 21 age- and sex-matched controls was selected from our samples (Table S1). The required sample size for this set was calculated based on the effect size of the result of the first set. The samples from the second set of participants were subjected to quantitative reverse transcriptase-polymerase chain reaction (qRT-PCR) analysis, as described later in detail. All eligible participants were diagnosed using the Diagnostic and Statistical Manual of Mental Disorders, Fourth Edition, Text Revision (DSM-IV-TR)^23^. Similar to our previous study^24^, the inclusion criteria for the FES group were as follows: age 15-40 years, no history of antipsychotic medications for psychosis for more than a total of 16 weeks at the time of registration, and continuous psychotic symptoms within the past 60 months. The exclusion criteria included the following: the presence or a history of other neurological illnesses, a traumatic brain injury with any known cognitive consequences or the loss of consciousness for >5 min, a history of electroconvulsive therapy, low premorbid IQ (<70), a history of alcohol addiction, previous continuous illegal substance use (e.g., cannabis) and the presence of clearly diagnosed autism spectrum disorders. For the control group, additional exclusion criteria included any current or previous history of psychiatric disease detected by screening with the modified Mini-International Neuropsychiatric Interview^25^. Patients in the FES group were evaluated using the Global Assessment of Functioning (GAF) scale^23^ and the Positive and Negative Syndrome Scale (PANSS)^26^.

**Table 1:** Demographic characteristics of exploration study participants

	Controls	FES	*P*-value^a^
Participants, *n*	17	17	1
Male	8	8	
Female	9	9	
Age (years), mean (SD)	24.9 (1.2)	23.5 (6.7)	0.49
DOI (weeks), mean (SD)	NA	39.3 (50.4)	NA
DUP (weeks), mean (SD)	NA	31.7 (52.1)	NA
GAF, mean (SD)	NA	37.5 (10.2)	NA
PANSS positive, mean (SD)	NA	15.1 (4.8)	NA
PANSS negative, mean (SD)	NA	18.6 (8.9)	NA
PANSS general psychopathology, mean (SD)	NA	34.4 (10.4)	NA
Chlorpromazine dose (mg/day), mean (SD)	NA	469.1 (513.8)	NA

*FES* first-episode schizophrenia, *DOI* duration of illness, *DUP* duration of untreated psychosis, *GAF* the global assessment of functioning, *NA* not applicable, *PANSS* the positive, negative, and general psychopathology scale scores, *SD* standard deviation

^a^Student *t*-test or chi-square test

This study was approved by the Medical Research Ethics Committee of the University of Tokyo Hospital (No. 2226 - [9], No. 2094 - [6] and 639 - [30]). Written informed consent was obtained from all participants.

### Blood sampling procedure

Peripheral blood samples were drawn by experienced physicians from a peripheral vein. The blood samples were collected between 1 PM and 3 PM after strict fasting for more than 3 h. Within 30 min of blood collection, plasma was isolated via centrifugation at 1200 × *g* for 10 min and then stored at −80 °C until use.

### Plasma miRNA expression profiling

Total RNA was extracted from 300 μl of plasma using the 3D-Gene RNA extraction reagent from a liquid sample kit (Toray, Kamakura, Japan) according to the manufacturer’s specifications. The extracted total RNA was labeled and hybridized onto 3D-Gene Human miRNA Oligo Chips (Toray), which are based on miRBase ver. 19, according to the manufacturer’s instructions. Data were analyzed using GeneSpring GX ver. 12.5 (Agilent Technologies, Santa Clara, CA, USA).

Global normalization was performed as follows: The raw signal data for each sample were log2-transformed, and the 75th percentiles of the expression values of each microarray were computed. These values were then subtracted from the expression value of each entity independently.

After normalization, the miRNAs for which the expression data were detected across all samples were extracted and subjected to statistical analyses.

### Plasma miRNA validation by qRT-PCR

MicroRNAs were extracted from 200 μl of plasma using the miRCURY RNA Isolation Kit - Biofluids (Exiqon, Vedbaek, Denmark) according to the manufacturer’s instructions. For the normalization of sample-to-sample variation, synthetic *Caenorhabditis elegans* miRNA-39 (cel-miR-39) was added to each denatured sample for qRT-PCR analysis. Subsequently, miRNA was transcribed to cDNA using the miScript II RT Kit (Qiagen, Hilden, Germany) and was detected by qRT-PCR using the miScript SYBR Green PCR Kit with a miScript Primer Assay (Qiagen). The relative miRNA expression level was determined by qRT-PCR cycle number with the levels normalized to the average cel-miR-39 transcript level using the ΔΔCT method^27^.

### Screening of miR-223 target genes using a combination of genome-wide gene expression and in silico analyses

Stable miR-223-overexpressing cells were selected by G418 (Wako Chemicals, Osaka, Japan) from SK-N-SH (human neuroblastoma cell line) cells (ATCC, Rockville, MD, USA) transfected with miR-223 overexpression plasmids (Cat# MI000030) or empty plasmid controls (Cat# pCMVMIR) purchased from OriGene (Rockville, MD, USA). After selection, three single clones from each group were randomly picked and maintained in DMEM with G418. To initiate differentiation, the cells were grown in DMEM containing 10 µM retinoic acid (RA) (Wako Chemicals, Osaka, Japan), 200 µl/ml G418 and 3% fetal bovine serum (FBS) in the dark; conditioned media were replaced every 72 h for 10 days.

Neuronal differentiation was morphologically confirmed and validated by Western blot analysis using antibodies against neuron-specific enolase (NSE) (#8171S; Cell Signaling Technology, Danvers, MA, USA), glial fibrillary acidic protein (GFAP) (G3893; Sigma-Aldrich, St Louis, MO, USA), and glyceraldehyde 3-phosphate dehydrogenase (GAPDH) (M171-7; MBL, Nagoya, Japan).

Subsequently, total RNA was extracted with the miRCURY RNA Isolation Kit - Cell & Plant (Exiqon). Using 100 ng of total RNA, biotin-labeled complementary RNA was synthesized and hybridized onto a SurePrint G3 Human GE 8 × 60 K v2 Microarray (Agilent Technologies) according to the manufacturer’s instructions. Data were analyzed using GeneSpring GX ver. 12.5 (Agilent Technologies). Briefly, as noted above, raw data were log2-transformed and normalized to the 75th percentile as recommended by the manufacturer. After normalization, only the genes with detection flags present in at least 50% of the samples were extracted and subjected to statistical analyses. To identify significantly downregulated mRNAs, the significance thresholds for unpaired t-tests were set to *P* < 0.05 and fold-change > 1.5.

For the in silico analysis, the target genes and their miRNA binding site seed regions were predicted using the miRWalk database (release 2.0, http://mirwalk.uni-hd.de/)^28^.

### Target mRNA validation by qRT-PCR

To validate the target genes of interest, i.e., those predicted by both the microarray and in silico analyses, qRT-PCR was conducted using transcript-specific primers designed as shown in Table S2. Total RNA was extracted from the cells immediately (day 0), 3, 6 and 10 days after RA treatment. Then, 500 ng of total RNA was transcribed into cDNA using the miScript II RT Kit (Qiagen) and was detected by the KAPA SYBR Fast qPCR Kit (Nippon Genetics, Tokyo, Japan). The relative mRNA expression level was determined by qRT-PCR, and the expression levels were normalized to the average GAPDH expression level using the ΔΔCT method.

### Plasmid construction and dual-luciferase reporter assay

The 3′ UTR fragments of the target genes of interest containing predicted miRNA binding sites were cloned into the dual-luciferase reporter vector pmirGLO (Promega, Madison, WI, USA) using the restriction enzyme pair NheI and SalI. To confirm specific binding to the predicted sites, we prepared another set of fragments with point mutations in the binding sites. These point mutations were introduced with the PrimeSTAR Mutagenesis Basal Kit (Takara, Otsu, Japan) following the provided protocol. The mutations were confirmed by sequencing. The primers used in cloning and mutagenesis are listed in Table S3. SK-N-SH cells were harvested 24 h after transfection and analyzed for firefly and Renilla luciferase activity using the Dual-Luciferase Reporter Assay System (Promega). Firefly luciferase activity values were normalized to Renilla luciferase activity levels. Three independent experiments were performed in triplicate.

### Statistical analysis

For the microarray analysis of the first set, statistical significance was assessed using Student’s *t*-test followed by the Bonferroni correction for multiple comparisons. For qRT-PCR analysis, the expression levels of the candidate miRNAs were compared between patients with FES and controls in the first set using a two-tailed *t*-test. In the second set, for the purpose of replicating the significant difference identified in the first set, a one-tailed t-test was utilized. Correlations between clinical symptoms and plasma miR-223 levels were assessed with Pearson’s correlation coefficients or linear mixed model analysis. For experiments using miR-223-OE cells, the statistical significance was determined by Student’s *t*-test for the microarrays and validation, by one-way analysis of variance (ANOVA) followed by Tukey’s honest significant difference test for luciferase assays, and by two-way ANOVA followed by the Bonferroni post hoc test for qRT-PCR analysis. Statistical analyses were carried out using R version 3.4.2 (R Foundation for Statistical Computing, Vienna, Austria), and a probability value of less than 5% was considered to indicate statistical significance.

## Results

### Demographic characteristics

Demographic characteristics of the subjects included in the first and second sets are shown in Tables 1 and S1, respectively. There were no significant differences in age or gender between patients with schizophrenia and the healthy control subjects in either set.

### Identification of the plasma miRNA signature

**Fig. 1:**
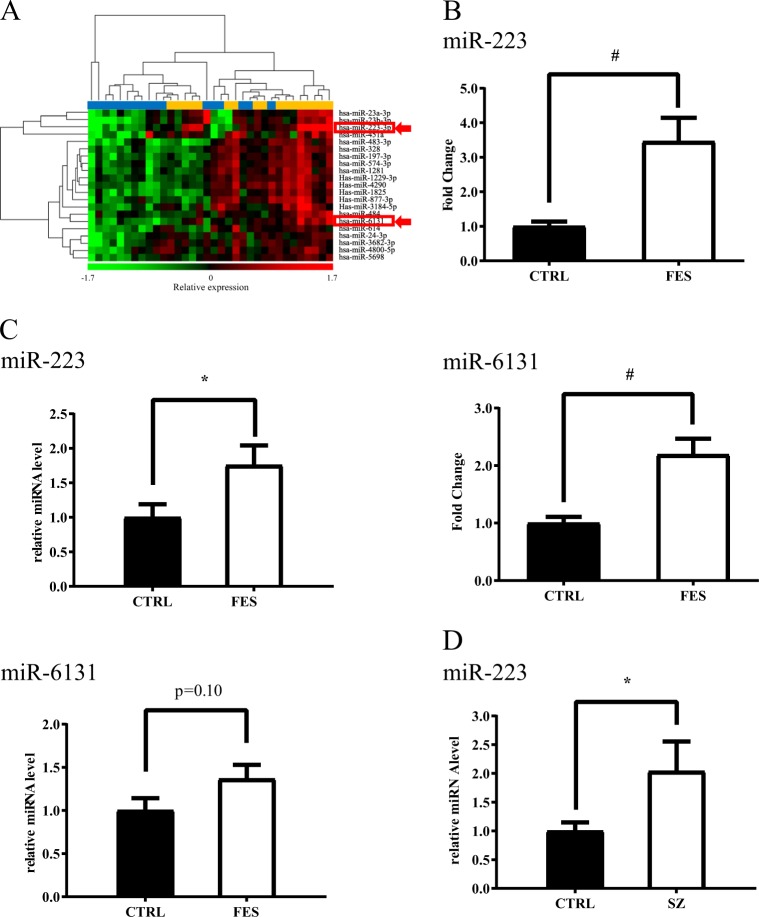
Identification and validation of elevated miR-223 in the plasma of first-episode schizophrenia. **a** Heat map showing the microarray expression data for 21 significantly altered miRNAs in plasma samples from patients with first episode of schizophrenia (FES, *n* = 17) and healthy control subjects (CTRL, *n* = 17). Statistical significance was analyzed using the unpaired *t*-test; *P* < 0.05 and fold change > 1.5 were considered to indicate statistical significance. MiRNA expression is hierarchically clustered on the *y*-axis, and the plasma samples from patients with FES or healthy control subjects are hierarchically clustered on the *x*-axis. The relative miRNA expression level is depicted according to the color scale shown at the bottom, which ranges from red (denoting high expression) to green (denoting low expression). The top horizontal bar indicates the controls in blue, and patients with FES are in orange. The miRNAs with expression levels that remained significantly higher in the FES group compared to the control group after Bonferroni correction (*P* < 0.05) are indicated with red arrows. **b** Microarray expression of select miRNAs. The microarray expression levels of miR-223 (upper panel) and miR-6131 (lower panel) are shown on a logarithmic scale (CTRL, *n* = 17; FES, *n* = 17). **c** Validation of miRNA microarray results by quantitative real-time PCR (qRT-PCR). The relative expression levels of miR-223 (upper panel) and miR-6131 (lower panel) were measured by qRT-PCR (CTRL, *n* = 17; FES, *n* = 15). **d** Validation of plasma miR-223 expression levels in patients with schizophrenia (SZ, *n* = 21) and controls (CTRL, *n* = 21). The validation sample size was calculated by the effect size with 80% power. The comparative threshold cycle (Ct) method was used with cel-miR-39 as an external control. The data presented are the average expression ± standard error of the mean. *P < 0.05 with respect to miRNA expression in control subjects, obtained via unpaired t-test. ^#^*P* < 0.05 with respect to miRNA expression in control subjects, obtained via unpaired *t*-test with Bonferroni correction. CTRL controls, FES first-episode schizophrenia, SZ schizophrenia

To investigate the possible involvement of plasma miRNAs in the initial stage of schizophrenia, the global miRNA expression in plasma was determined by 3D-Gene Human miRNA Oligo Chips from 17 patients with FES and 17 healthy controls. Twenty-one miRNAs were identified as differentially expressed (fold change > 1.5 and adjusted *P*-value < 0.05) between patients with FES and controls (Fig. 1a). Among them, hsa-miR-223-3p (miR-223) and hsa-miR-6131 (miR-6131) remained significant after Bonferroni correction (Fig. 1b).

### Validation of elevated miR-223 and miR-6131 expression

To validate the alterations in these two miRNAs, a qRT-PCR analysis was performed in two steps. First, plasma miRNA expression was measured from the same sample set using a different miRNA extraction method. Given that the plasma samples from two patients with FES were used in the microarray analysis, qRT-PCR was conducted with samples from 15 patients with FES and 17 controls. The relative expression level of miR-223 was significantly higher (1.8-fold) in patients with FES than in controls (*t*[30] = 2.23, *P* *=* 0.03, Fig. 1c, upper panel), while the relative expression level of miR-6131 showed a 1.4-fold increase (*t*[30] = 1.68, *P* *=* 0.10, Fig. 1c, lower panel). Second, according to the effect size of the relative miR-223 expression level (Cohen’s *d* = 0.79), the required sample size was calculated. As a result, 21 patients with schizophrenia (an average of 7.0 years after the onset of the disease) and 21 healthy age- and sex-matched controls were subjected to qRT-PCR analyses, as described in the methods section. The upregulation of miR-223 expression in patients with schizophrenia was confirmed in the replication sample set (*t*[40] = 1.93, *P* *=* 0.03, Fig. 1d). To assess correlations between the relative plasma level of miR-223 and clinical parameters, including chlorpromazine dose and the results of clinical scales, such as PANSS and GAF, we performed a correlation analysis using Pearson’s correlation coefficients. In FES patients, the miR-223 level was significantly correlated with the duration of illness (DOI) (Pearson’s *r* = −0.57, *P* *=* 0.03), while this correlation was not significant in schizophrenia patients in the second set (Pearson’s *r* = 0.28, *P* *=* 0.22, Table S4). Linear mixed model analysis using the data from both sets revealed that the miR-223 level in all patients had no significant correlation with the DOI. There was no significant correlation between the plasma miR-223 level and any other clinical parameter (Table S4).

### Identification of putative genes targeted by miR-223

**Fig. 2:**
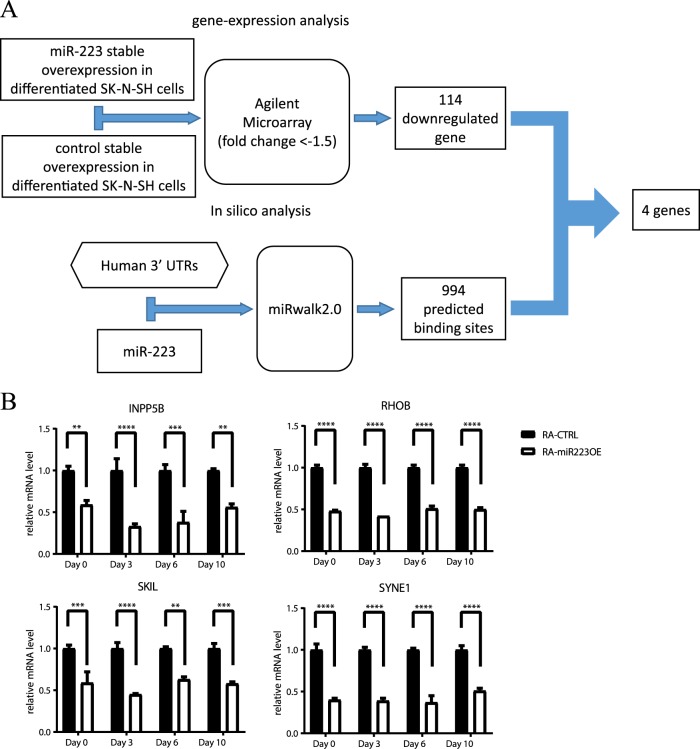
Evaluation of miR-223 target genes. **a** miR-223 target prediction method. To identify novel miR-223 target genes, a genome-wide gene expression analysis and an in silico analysis were performed. First, the gene expression analysis was performed using differentiated SK-N-SH cells with stable miR-223 overexpression (*n* = 3), which were compared to empty vector controls (*n* = 3). A total of 114 downregulated (fold change < −1.5) genes were selected as putative targets of miR-223. Next, miR-223 and the entire 3′ UTR of the human genome were used as inputs for the miRNA target prediction program, miRWalk2.0. A set of 994 binding sites was predicted to be targeted by miR-223 by at least one prediction program. Finally, there was an overlap of four genes between the gene expression and in silico analyses. UTR, untranslated region. **b** Quantitative RT-PCR validation of putative miR-223 target gene expression. The relative expression levels of *INPP5B* (upper left), *RHOB* (upper right), *SKIL* (lower left) and *SYNE1* (lower right) were confirmed by qRT-PCR. The comparative threshold cycle (Ct) method was used with GAPDH as an internal control. The data represent the average expression ± standard error of the mean (*n* = 3, each group). **P *<* 0.01, ***P < 0.001, ****P < 0.0001, with respect to control SK-N-SH cells determined by two-way ANOVA, followed by the Bonferroni post hoc test. RA Retinoic acid, OE overexpression, CTRL control

To further explore the putative target genes of miR-223, an integrative gene expression analysis and an in silico data analysis were performed (Fig. 2a). First, stable clones overexpressing miR-223 (miR-223-OE cells) and empty controls were selected with G418. After a 10-day treatment with 10 μM RA, most of the cells acquired a neuronal morphology (Fig. S1A). Additionally, the neuronal differentiation of neuroblastoma cells was confirmed by Western blotting using antibodies against NSE, GFAP and GAPDH (Fig. S1B). qRT-PCR analysis confirmed that the expression level of miR-223 in miR-223-OE cells was continuously increased compared to that in empty vector control cells 0, 3, 6 and 10 days after RA treatment (Fig. S2). Two-way ANOVA showed no correlation between genotype and time. Microarray analysis revealed that 247 probe sets were differentially expressed with fold change > 1.5 and *P* < 0.05 compared with the empty vector control. Since it is known that miRNAs bind to specific target mRNAs and downregulate their transcription, a certain number of downregulated mRNAs might be the consequence of the direct effect of miR-223 binding to target sites. Therefore, based on the results of the gene expression analysis, 114 probe sets that were downregulated in miR-223-OE cells were selected as putative targets. Meanwhile, for the in silico analysis, miR-223 and the entire 3′ UTR of the human genome were used as inputs for the miRNA target prediction program miRWalk2.0. A set of 994 binding sites was predicted to be targeted by miR-223. Finally, four genes, namely, type II inositol polyphosphate 5-phosphatase (*INPP5B*), Ras homolog family member B (*RHOB*), SKI-like proto-oncogene (*SKIL*) and spectrin repeat containing, nuclear envelope 1 (*SYNE1*), were defined as candidate target genes because they were detected in both the gene expression and in silico analyses.

### Validation of the putative target genes of miR-223

To validate the suppression of putative target mRNA expression in miR-223-OE cells, qRT-PCR was conducted to quantitatively evaluate the synthesis of these mRNAs. The expression levels of the four selected mRNAs mentioned above (INPP5B, RHOB, SKIL, and SYNE1) were significantly decreased before neuronal maturation (day 0), and a significant decrease was also observed 3, 6, and 10 days after RA treatment. Two-way ANOVA showed no interaction between genotype and time after RA treatment. These results are comparable to those obtained from the microarray analysis (Fig. 2b).

**Fig. 3:**
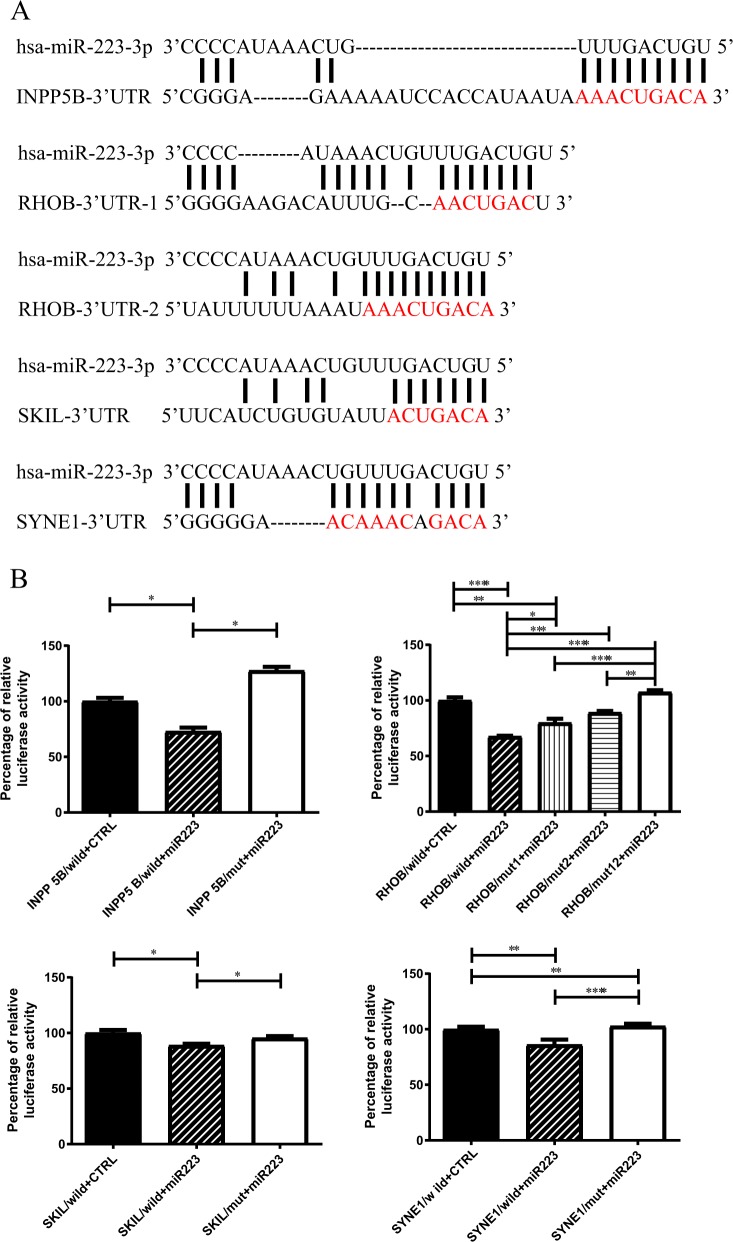
Validation of downstream miR-223 target genes. **a** Alignment of the predicted miR-223 binding sites in the *INPP5B*, *RHOB*, *SKIL* and *SYNE1* 3′ UTRs. The sites of targeted mutagenesis are indicated in red. Note that *RHOB* has two binding sites, as indicated in a previous report. (**b**) Dual-luciferase reporter assay for the validation of the miR-223 target sites in the 3′ UTRs of *INPP5B* (upper left), *RHOB* (upper right), *SKIL* (lower left), and *SYNE1* (lower right). The wild-type 3′ UTR sequences of the 4 predicted genes and the sequences with mismatched mutants were cloned into pmirGLO luciferase reporter vectors. The wild-type (wild) or mutated (mut) 3′ UTRs were cotransfected with miR-223 or scrambled (CTRL)-overexpression plasmids in SK-N-SH cells, and the luciferase assay was subsequently performed. The data show the average percentages of firefly and Renilla luciferase activity ± standard errors of the mean (*n* = 3, each group). The groups were compared by ANOVA, followed by Tukey’s HSD post hoc test; *P < 0.05, **P < 0.01, ***P *<* 0.001, ****P < 0.0001

According to the prediction algorithms used in the current study, miR-223 has one putative binding site each in *INPP5B*, *SKIL* and *SYNE1*; however, in *RHOB*, there are two binding sites, which have previously been reported^29^ (Fig. 3a). To evaluate the direct interaction between miR-223 and these target sites, a luciferase reporter assay was performed using the pmirGLO Dual-Luciferase miRNA Target Expression Vector. For each gene, the section of the 3′ UTR containing the miRNA binding site was cloned into a pmirGLO vector. To confirm whether miR-223 truly targets these specific sites, another set of sequences with point mutation of the binding sites was cloned. SK-N-SH cells were transfected with either a wild-type or a mutated construct, along with miR-223 or the scrambled overexpression plasmid. Our results revealed that the luciferase activities of the wild-type *INPP5B*, *RHOB*, *SKIL*, and *SYNE1* 3′ UTR luciferase reporters were significantly attenuated in SK-N-SH cells cotransfected with miR-223 compared to SK-N-SH cells cotransfected with a scrambled control. Furthermore, the luciferase activities of the constructs with mutated binding sites were not affected by miR-223 (Fig. 3b).

## Discussion

MiRNAs have emerged as important regulators that are involved in brain development and function by controlling the expression of various genes, and these miRNAs can potentially contribute to an individual’s susceptibility to schizophrenia^30^. Evidence from recent studies using postmortem brains or peripheral blood samples supports the association between altered miRNA expression and schizophrenia^15–17,31,32^. However, regarding the interpretation of altered miRNA expression in postmortem brains, it is difficult to distinguish the effects of aging and long-term medication use from the effects of the disease itself^33,34^. Given that circulating miRNAs, unlike other RNAs, have been reported to exhibit an unexpected level of stability, disease-specific changes in circulating miRNA expression have become a research focus^22,35,36^. Although the origin and function of circulating miRNAs are largely unclear, emerging evidence suggests that they might serve as biomarkers for various diseases and may be signaling molecules associated with intercellular communication^37,38^. Hence, the detection of schizophrenia-related alterations in circulating miRNA levels may contribute to a better understanding of the potential role of miRNA in this disorder. Furthermore, considering the aforementioned involvement of miRNAs in the regulation of neural development, it is important to investigate the early stages of schizophrenia, including disease onset, which has the benefit of excluding the effects of long-term medication use, a potential confounding factor. Overall, in this study, we attempted to detect changes in miRNA expression levels in plasma from patients with FES and to subsequently analyze the possible target mRNAs, which could reveal the molecular characteristics related to the onset of schizophrenia.

In the present study, global expression profiling of plasma miRNAs revealed the differential expression of various miRNAs. Among them, miR-223 was significantly upregulated in patients with FES. This result was validated by qRT-PCR using a different RNA extraction method. The significant elevation of miR-223 was replicated in an independent cohort of patients with the later stage of schizophrenia, assuming a continuous increase in plasma miR-223 levels at disease onset and at later stages. MiR-223 was first identified in the hematopoietic system^39,40^; however, recent studies have indicated a possible role of miR-223 in psychiatric disorders, such as schizophrenia. Notably, a postmortem brain study in patients with schizophrenia showed that miR-223 was one of 33 miRNAs that were upregulated in the dorsolateral prefrontal cortex^11^. Although there are only a handful of studies describing the function of miR-223 in the brain, a recent study reported that miR-223 has a neuroprotective effect by regulating the expression and function of glutamate receptors^41^. The antipsychotic drug olanzapine has been reported to downregulate the expression of miR-223 in the mouse brain^34^. However, since our study showed no correlation between the expression of miR-223 and chlorpromazine dose equivalents (Table S4), the elevated miR-223 level was not due to the effects of medication during the first episode. We also found that the plasma level of miR-223 in the first set of FES patients was negatively correlated with the DOI; in contrast, there was no such correlation at the later stage in the second set of patients. In this study, as described in the materials and methods section, one of the inclusion criteria for FES regarding the DOI was continuous psychotic symptoms within the past 60 months. However, the actual average DOI in the first set with FES was less than 1 year (39.3 ± 50.4 weeks), whereas the average in the second set was ~7 years (369.0 ± 244.4 weeks), suggesting different populations. Therefore, these results could be interpreted as the elevated expression of miR-223 around the onset of disease that subsequently declined over the next several years, most likely stabilizing at a later stage. Combining previous results with our present data, it could be suggested that miR-223 is involved in the pathophysiology of schizophrenia, at least in a specific subset of the patient population.

Based on an integrative analysis using both in silico target prediction and the evaluation of gene expression in miR-223-OE cells, four putative target genes, *INPP5B*, *RHOB*, *SKIL*, and *SYNE1*, were detected. Our results show that, under conditions of miR-223 overexpression, the expression levels of these four genes were consistently decreased, regardless of the differentiation state of the cultured cells. The miRNA-mRNA binding of these four target genes was further confirmed by a luciferase reporter assay. With the exception of *RHOB*, which had already been reported to have two binding sites for miR-223 in the 3’ UTR^29^, three miR-223 target mRNAs (*INPP5B*, *SKIL* and *SYNE1*) were newly identified in this study.

*INPP5B* is involved in the endocytic and early secretory pathways by regulating the trafficking of biosynthetic cargo, which is strongly related to Lowe syndrome^42,43^. One of the neural symptoms of Lowe syndrome is myelin dysfunction, which also contributes to the neural abnormalities in schizophrenia^44–46^. In the brain, *RHOB*, a member of the rho family of GTP-binding proteins, plays a regulatory role in synaptic plasticity and in the migration of neural cells^47,48^. *SKIL*, also known as *SNON*, is the negative corepressor of the TGF-beta signaling pathway in primary granule neurons^49^. Previous studies have demonstrated that in postmitotic neurons, *SKIL* regulates neuron branching, migration and positioning via the modification of axon development, migration, and dendrite morphogenesis^50–52^. In addition, *SYNE1*, also known as Nesprin 1, is associated with the regulation of cell shape and migration by linking the nucleus to the cytoskeleton^53,54^. According to a large-scale exome-wide association analysis of schizophrenia, *SYNE1* is a high risk gene^55–58^. It is striking that these four genes are related to neuronal migration and/or development and are known to be related to the pathophysiology of schizophrenia.

Animal models of schizophrenia have been generated in previous studies by manipulating genes related to the Neuregulin1, Disc1 and 22q11.2 deletion syndrome; all animal models showed neuronal migration abnormalities, which caused neuronal dysfunction similar to that observed in patients with schizophrenia^59^. In particular, it has been suggested that the disruption of miRNA-mediated gene regulation may be involved in schizophrenia-related phenotypes in an animal model of 22q11.2 deletion syndrome.

To our knowledge, no other studies have reported a significant change in plasma miR-223 levels in schizophrenia. Interestingly, however, several reports using other methods have revealed the abnormal expression of miRNAs, such as miR-130b, miR-193a-3p and miR-34a, which are related to neuronal migration and/or neural development, in the plasma of patients with schizophrenia^22,60^. For example, miR-34a regulates newborn neuronal function by downregulating several synaptic proteins and receptor subunits and reducing the migration of neuroblasts in vivo^61^. These results also suggest a possible relationship between the altered expression of miRNAs and the neurobiological changes underlying schizophrenia.

Taken together, the data suggest the hypothesis that miR-223 may play a role in neural development and neuronal migration via the downstream regulation of genes, which could, in turn, contribute to the etiology of schizophrenia.

However, the present study has several limitations that need to be acknowledged. First, the present sample size is small, and the replication sample set did not have patients with FES. In this study, as noted above, the average DOI for the first set of patients with FES was <1 year, whereas the average DOI for the second set of patients was ~7 years, suggesting that these two sets are different populations. In addition, due to the lack of samples from patients in remission, we did not evaluate state-dependent changes in the expression of miR-223. Thus, it is necessary for further studies to be conducted on a larger scale, including more patients with FES. In fact, the abnormal expression of plasma miRNA in patients with schizophrenia has been intensively investigated, especially in recent years^21,22^. Nevertheless, the results of these studies, including those of the present study, are inconsistent with one another. These discrepancies may be due to differences between sample size or population examined or the application of different technological platforms. Therefore, as noted above, the involvement of miR-223 might be applicable to only a specific subset of patients with schizophrenia. Second, this study used only miR-223-OE cells and did not show a clear relationship between miR-223 and glutamate signaling, although an increase in NR2B and GluR2 levels was previously shown in the hippocampus of miR-223-deficient mice^41^. Although glutamate receptors are strongly expressed in differentiated SK-N-SH cells^62^, we were not able to detect any alterations in these receptors. This discrepancy may be due to a difference in signal alterations induced by the deficiency or overexpression of miR-223. Another reason for this finding may be the differences between the analyzed cell types. Nonetheless, research using animal models overexpressing miR-223 will be required to further address this issue.

In conclusion, through a global expression analysis of plasma miRNA from patients with FES, the present study demonstrates the upregulation of miR-223. Subsequent experimental validation in miR-223-OE cells and an in silico prediction revealed the novel mRNA targets of miR-223: *INPP5B*, *RHOB*, *SKIL*, and *SYNE1*. Abnormal miR-223 levels may provide clues to the pathophysiology of schizophrenia.

## Supplementary information

Supplemental Materials
