# Supplementary material for: Altered expression of microRNA-223 in the plasma of patients with first-episode schizophrenia and its possible relation to neuronal migration-related genes
Source: Transl Psychiatry. 2019 Nov 11;9:289. doi: 10.1038/s41398-019-0609-0 (PMC6848172; doi:10.1038/s41398-019-0609-0)
Supplement: Supplementary file 1 — Supplemental Materials [file 41398_2019_609_MOESM1_ESM.pdf]

Zhao Z et al.

Supplemental Materials

Figure S1: RA induces neuronal cell differentiation.

A)

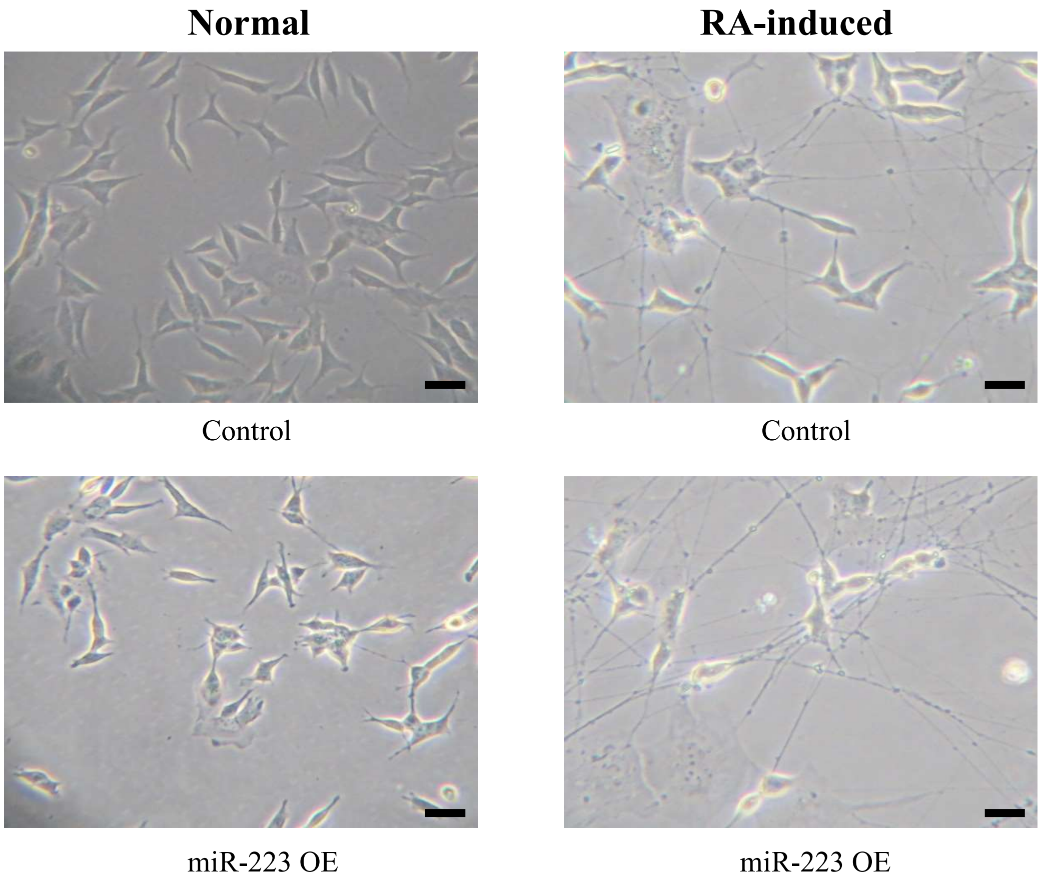

B)

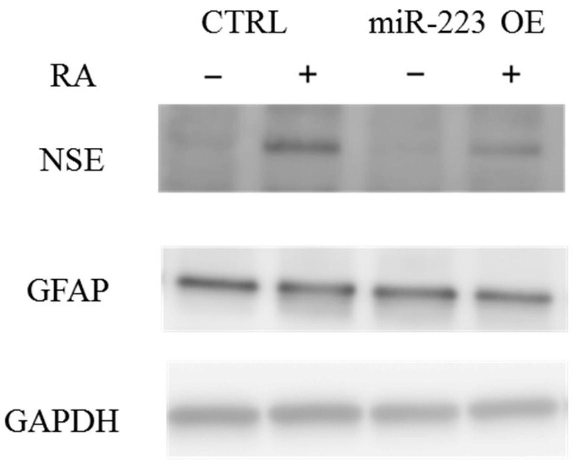

A) Phase contrast microscopy was used to evaluate the morphological features of miR-223-OE cells, which were differentiated in neuronal cells, following treatment with RA for 10 days.

B) Expressions of NSE and GFAP were analyzed by western blot. GAPDH was used as a loading control.

RA, retinoic acid; OE, overexpression; NSE, Neuron-specific enolase.

Figure S2: Validation of miR-223 overexpression by qRT-PCR.

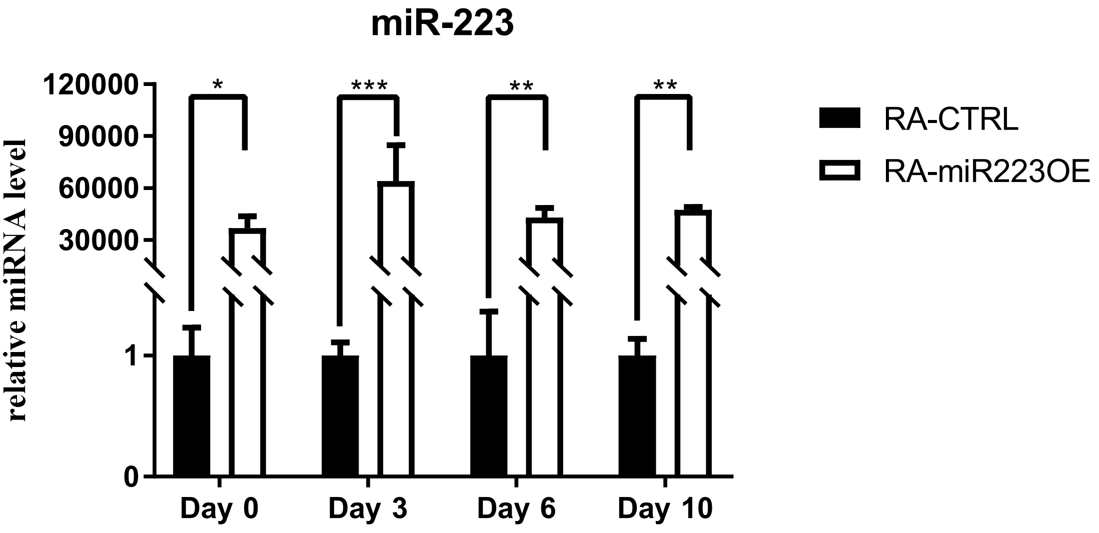

The comparative threshold cycle (Ct) method was used for quantification of miR-223 expression. RU6B was used as an internal control. The data presented indicate average expression values ± the standard error of the mean (n = 3, each group). Groups were compared by a two-way ANOVA (time x treatment) with a post-hoc Bonferroni multiple comparison test; *P < 0.05, **P < 0.01, ***P < 0.001. RA, Retinoic acid-treated; OE, overexpression; CTRL, control.

Table S1: Demographic characteristics of replication study participants

	Controls	*SZ*	P-*value*
Participants, n	21	21	1.00[a]
Male	10	10	
Female	11	11	
Age (years), mean (SD)	30.3 (3.3)	30.0 (5.4)	0.86[b]
DOI (weeks), mean (SD)	NA	369.0 (244.4)	NA

Abbreviations: DOI, duration of illness; SZ, schizophrenia; NA, not applicable; SD, standard deviation. *P* values were determined by chi-square test[a] or Student's t-test[b].

Table S2: qPCR primer sequences used in the study

Gene		primer sequence
GAPDH	sense	AAAGGGTCATCATCTCTG
	antisense	GCTGTTGTCATACTTCTC
INPP5B	sense	AATGTCTTCCACTACTTG
	antisense	CTTCTTCTTCTCTGTCATA
RHOB	sense	TAGCATTCTGACCACACT
	antisense	GCCACATTCTTCAAGGAA
SKIL	sense	CCTCAATATAGATACCTCACTT
	antisense	AAGACTACTATGTTGGCTTAA
SYNE1	sense	GTCTACGATTCCTGTAACTC
	antisense	TGTGCTTCTCTATGTCTCT

Table S3: Primer sequences for miRNA target validation used in this study

Gene		Primer sequence (5'-3')	Use
INPP5B	sense	TAGGCTAGCTGTGAGTCGTGAAGACACGG	Cloning of 3' UTR of *INPP5B* into pmirGlo
	antisense	CTAGTCGACCCTGGAGGTAGGGTGAGTGA	
RHOB	sense	TAGGCTAGCCCCATCCCAGTGTCTGTGTG	Cloning of 3' UTR of *RHOB* into pmirGlo
	antisense	CTAGTCGACGCTCACCATTTTGTCATTTGTCAG	
SKIL	sense	TAGGCTAGCCTGTTAAAGAGATTCATCTG	Cloning of 3' UTR of *SKIL* into pmirGlo
	antisense	CTAGTCGACAACTGCTGCTTCTGTTTAGCA	
SYNE1	sense	TAGGCTAGCTCTGCAGAAGTGCTGGTAGC	Cloning of 3' UTR of *SYNE1* into pmirGlo
	antisense	CTAGTCGACGAAAGCTGCCCAGATGGTCT	
INPP5B mut	sense	TAGGGAGAGTGTTCAATTTTCAACTTTAGTTAT	Generating the point mutations on the binding site of *INPP5B*
	antisense	TTGAACACTCTCCCTATTATGGTGGATTTTCTC	
RHOB mut1	sense	TTGCCTAGTCATTGGGGAGGACACAGCTT	Generating the point mutations on the binding site of *RHOB*
	antisense	CCAATGACTAGGCAAATGTCTTCCCCAGG	
RHOB mut2	sense	TTTAACGCGCTATGACAATGACAAAATGGTGAG	Generating the point mutations on the binding site of *RHOB*
	antisense	TCATTGTCATAGCGCGTTAAAAAATAAAAAACAAG	
SKIL mut	sense	GTATTTGACACAAGGTTTTTTTGTTTG	Generating the point mutations on the binding site of *SKIL*
	antisense	AAAACCTTGTGTCAAATACACAGATGAA	
SYNE1 mut	sense	CGGGGGAGTCGGTATCAGACAAGAAGGTTTGGAA	Generating the point mutations on the binding site of *SYNE1*
	antisense	TCTTGTCTGATACCGACTCCCCCGTCACTGTTTA	

Table S4 Correlations between relative level of miR-223 and clinical assessment

	Relative level of miR-223				
	First set		*Second set*		*Total (First & Second sets)*
	Pearson's *r*	*P-value*[a]	Pearson's *r*	*P-value*[a]	*P-value*[b]
Age	0.35	0.20	0.18	0.44	NA
logDOI (weeks)	-0.57	0.03*	0.28	0.22	0.18
logDUP (weeks)	-0.46	0.08	NA	NA	NA
GAF	0.21	0.45	NA	NA	NA
PANSS positive	-0.38	0.17	NA	NA	NA
PANSS negative	0.09	0.75	NA	NA	NA
PANSS general psychopathology	0.09	0.75	NA	NA	NA
Chlorpromazine dose	0.08	0.77	NA	NA	NA

Abbreviations: DOI, duration of illness; DUP, duration of untreated psychosis; GAF, the global assessment of functioning; NA, not applicable; PANSS, the positive, negative, and general psychopathology scale scores. *P* values were determined by [a]two-tailed Pearson's correlation or [b]linear mixed model. *$P < 0.05$
